# "Rate My Therapist": Automated Detection of Empathy in Drug and Alcohol Counseling via Speech and Language Processing

**DOI:** 10.1371/journal.pone.0143055

**Published:** 2015-12-02

**Authors:** Bo Xiao, Zac E. Imel, Panayiotis G. Georgiou, David C. Atkins, Shrikanth S. Narayanan

**Affiliations:** 1 Department of Electrical Engineering, University of Southern California, Los Angeles, United States of America; 2 Department of Educational Psychology, University of Utah, Salt Lake City, United States of America; 3 Department of Psychiatry and Behavioral Sciences, University of Washington, Seattle, United States of America; Tokai University, JAPAN

## Abstract

The technology for evaluating patient-provider interactions in psychotherapy–observational coding–has not changed in 70 years. It is labor-intensive, error prone, and expensive, limiting its use in evaluating psychotherapy in the real world. Engineering solutions from speech and language processing provide new methods for the automatic evaluation of provider ratings from session recordings. The primary data are 200 Motivational Interviewing (MI) sessions from a study on MI training methods with observer ratings of counselor empathy. Automatic Speech Recognition (ASR) was used to transcribe sessions, and the resulting words were used in a text-based predictive model of empathy. Two supporting datasets trained the speech processing tasks including ASR (1200 transcripts from heterogeneous psychotherapy sessions and 153 transcripts and session recordings from 5 MI clinical trials). The accuracy of computationally-derived empathy ratings were evaluated against human ratings for each provider. Computationally-derived empathy scores and classifications (high vs. low) were highly accurate against human-based codes and classifications, with a correlation of 0.65 and F-score (a weighted average of sensitivity and specificity) of 0.86, respectively. Empathy prediction using human transcription as input (as opposed to ASR) resulted in a slight increase in prediction accuracies, suggesting that the fully automatic system with ASR is relatively robust. Using speech and language processing methods, it is possible to generate accurate predictions of provider performance in psychotherapy from audio recordings alone. This technology can support large-scale evaluation of psychotherapy for dissemination and process studies.

## Introduction

Service delivery data [1] indicate that millions of Americans are receiving treatment for substance abuse, many of whom are attending psychotherapy. Although the research literature points to efficacious psychotherapies (e.g., motivational interviewing; MI;[2]), research-based training and supervision procedures–with small numbers of therapists, intensive training, and ongoing supervision using performance-based feedback [3]–will never translate to real-world settings. Thus, the quality of psychotherapy in clinical practice is largely unknown.

Unlike pharmaceuticals, where quality is monitored during the manufacturing process and regulated by the Food and Drug Administration, the quality of psychotherapy is unknown until the patient and provider interact. As a result, both quality assurance and treatment mechanism research rely on human evaluation of patient-provider interactions. The gold standard for evaluating psychotherapy is observational coding–humans listening to and identifying relevant behaviors and language according to some theoretically derived criteria–a procedure first formally used by Carl Rogers in the 1940s. [4] Observational coding of psychotherapy has provided important insights into the process and outcome of psychotherapy (e.g., [5]), but it is time consuming, expensive, and introduces privacy concerns.

In contrast, technological innovation has dramatically influenced other aspects of mental health science. [6] Imaging techniques have documented the effects of psychotherapy on the brain [7], and computerized adaptations of psychotherapies now have the potential to augment human providers. [8] In contrast, human evaluation of psychotherapy via observational coding–the core technology behind measures of patient and provider interactions–has remained fundamentally unchanged for 70 years, hindering the large-scale evaluation of treatments in the community. A scalable mechanism for evaluating psychotherapy process is needed.

### Motivational Interviewing

Among the many evidence-based psychotherapies currently available, MI [2] provides a clear example of the challenges with observational coding. MI includes a broad class of interventions in which the counselor maintains an empathic, nonjudgmental stance while using specific linguistic techniques (e.g., reflections) to promote client “change talk” (i.e., verbal statements about changing the identified behavior). The efficacy of MI is supported by numerous clinical trials, and treatment mechanisms are well specified. [9] There is an international organization devoted to the training and dissemination of MI (The Motivational Interviewing Network of Trainers [MINT]), and MI developers have been at the forefront of research on training and dissemination. Yet, even with this accumulated knowledge and organizational support, community counselors learning MI require ongoing, performance-based feedback via observational coding (called provider fidelity) for counselors to maintain proficient MI skills. [10]

Unfortunately, coding requires training raters, as well as establishing and maintaining inter-rater reliability. Including training time, coding a 20 minute segment of MI can require 85 minutes (roughly 4:1), and a somewhat higher estimate suggested 90–120 minutes per 20 minute segment (5–6:1) [11]–and this cost does not scale up as more sessions are coded due to concerns with coder turnover (e.g., some coders quit or graduate and must be replaced) and the tendency for coders to drift away from reliability after initial training. As the actual number of sessions for drug and alcohol abuse in the U.S. are likely in the millions [1], observational coding of provider fidelity will never be adopted as a widespread quality improvement tool. [12]

### Behavioral Signal Processing

Behavioral signal processing (BSP) [13] is an emerging field in engineering and computer science that utilizes computational methods to assist in human decision-making about complex behavioral phenomena. Essentially, it is an attempt to quantify the “felt sense” of expressed human behavior. There is initial evidence that semantic information from human-generated transcripts of therapy sessions can be used with modern, text-based machine learning models to accurately predict a range of observational codes. [14–16] On the one hand these works are encouraging, as they demonstrate that it is possible to quantify the semantic signal in behavioral coding of psychotherapy, but they still rely on human transcription, which is labor intensive and unrealistic in the real-world.

Using an audio recording of a therapy session to automatically generate behavioral codes without human intervention presents several speech signal processing challenges. Sessions typically involve two speakers, and speaking turns need to be automatically segmented and associated with their identity; moreover, these speakers may overlap and interrupt one another during a therapy session posing additional difficulties for the automated processing. Advances in audio signal “diarization” (i.e., determining who spoke when) and speech recognition (i.e., discerning what was said) [17–22] have brought such a technology for automating behavioral coding within reach. These engineering advances have led to tools to combine various pieces of information (e.g., from voice, language, interaction patterns, affect) that can be gleaned from session audio into predictive models of human-defined knowledge (e.g., empathy codes).

### Summary and Current Proposal

Up to the present time, assessing the quality of psychotherapy (i.e., provider fidelity) is synonymous with observational coding by humans, which is time consuming, expensive, and requires extensive training to maintain adequate reliability. Accordingly, there is no scalable method for assessing the content of psychotherapies like MI that are thought to rely on the content of specific linguistic exchanges between patients and providers. Moreover, what is said during the actual clinical encounter of psychotherapy and its relationship to patient outcome is largely a mystery.

Signal processing methods and computational models of psychotherapy process have the potential to dramatically reduce feedback delays and thus may “scale up” to large evaluation tasks (e.g., thousands of sessions a year in a busy clinic) that are currently unfeasible because of labor-intensive human evaluations. The primary contribution of the current paper is the following: the development of a behavioral signal processing (BSP) approach for evaluating provider empathy in MI, and leveraging speech and language processing techniques (e.g., automatic speech recognition and speaker identification) to process audio recordings of sessions for use with predictive text models that detect counselor empathy.

## Method

### Data sources: Psychotherapy Corpora

Three separate collections of psychotherapy sessions were used for different purposes in the present analyses. One collection included the focal MI sessions, in which counselor empathy was predicted. The other two collections served key functions in informing the speech signal processing tasks. An overview of the psychotherapy corpora used in this study is provided in Table 1. This study represents a secondary data analysis of archived recordings of treatment encounters and thus cannot be fully anonymized. All research procedures for this study were reviewed and approved by Institutional Review Boards at the University of Washington (IRB_36949) and University of Utah (IRB_00058732). During the original trials all participants provided written consent. The UW IRB approved all consent procedures.

**Table 1:** Summary of psychotherapy corpora and role in automatic empathy evaluation.

	# Session	# Talk turn	# Words	Has audio	Usage
General psychotherapy	1205	300863	6550270	No	ASR-LM training
MI Randomized Trials ^a^	153	36907	1123842	Yes	ASR-AM & ASR-LM training
CTT Trial ^b^	200	23985	624395	Yes	Empathy Detection

*Note*. ASR = Automatic speech recognition; LM = language model; AM = acoustic model

^a^ For further information on the specific MI randomized trials are summarized see. [24] Specific studies include Alcohol Research Collaborative: Peer Programs; [52] Event Specific Prevention: Spring Break; [53] Event Specific Prevention: Twenty First Birthday; [54] Brief Intervention for Problem Drug Use and Abuse in Primary Care; [55] Indicated Marijuana Prevention for Frequently Using College Students. [56]

^b^ CTT = Context Tailored Training. [23]

#### Dataset for empathy prediction (Context Tailored Training; CTT)

Empathy prediction was evaluated from sessions that were rated as having either “high” or “low” counselor empathy (n = 121 high empathy, n = 79 low empathy). The original study was a multi-site randomized trial comparing a specific form of training for substance abuse counselors in MI. Counselors were located at clinics affiliated with National Institute of Drug Abuse (NIDA) Clinical Trials Network (CTN) community substance abuse treatment facilities in the state of Washington (Context Tailored Training; CTT). [23] Counselors received approximately 15 total hours of time with MI trainers in either training conditions. Counselors were evaluated with six MI treatment sessions conducted with both standardized patients (SPs) and real patients (RPs) prior to training, post-training, and at 3-month follow-up. Each session was 20 minutes in total length.

Therapists from CTT were 68% female, and 72% Caucasian, 10% African American, 7% Native American, 6% Hispanic/Latino, 3% Asian, and 2% Pacific Islander. Mean age was 46.4 years (SD = 11.5). On average, they had provided clinical services for 9.5 years (SD = 8.7), and 25% had a master’s degree, 4% had a doctoral degree, 27% had a bachelor’s degree, 29% had an associate’s degree, and 14% had a high school diploma or equivalent. Most counselors had prior exposure to MI (61%), but in different ways. For example, 11% had participated in prior workshops, and 50% had read MI texts or journal articles prior to study trainings (see [23] for additional information on the sample).

#### Datasets for training Automatic Speech Recognition (ASR)

Two additional collections of psychotherapy sessions were used in training speech signal processing models. First, a large collection of 1,200 psychotherapy transcripts were used to help define the typical vocabulary and language use patterns of psychotherapy sessions (see [15] for more details on the corpus as well as the technical supplement (S1 File) and below for an overview of the speech recognition process). These sessions come from the implementation of a variety of theoretical approaches (e.g., MI, cognitive-behavioral therapy, psychoanalysis, etc.) and have a variety of clinical foci (e.g., anger, depression, suicide, substance abuse, etc.).

A smaller collection of session transcripts (n = 153) were randomly sampled from five MI clinical trials (out of 899 total sessions [24]). This collection was similarly used for helping to define the common language use in MI based psychotherapy for the speech recognition task. In addition, because audio recordings were available, this collection helped train acoustic models (i.e., of how phones sound) in the ASR process. In our ASR system, phonemes are small sound units, roughly corresponding to letters that make up words (e.g. “W ER D” represents “WORD”). The ASR system recognizes phones, parses the phones into words, and concatenates the words into utterances.

### Measures

#### Motivational Interviewing Treatment Integrity 3.0 (MITI3.0)

**Fig 1:**
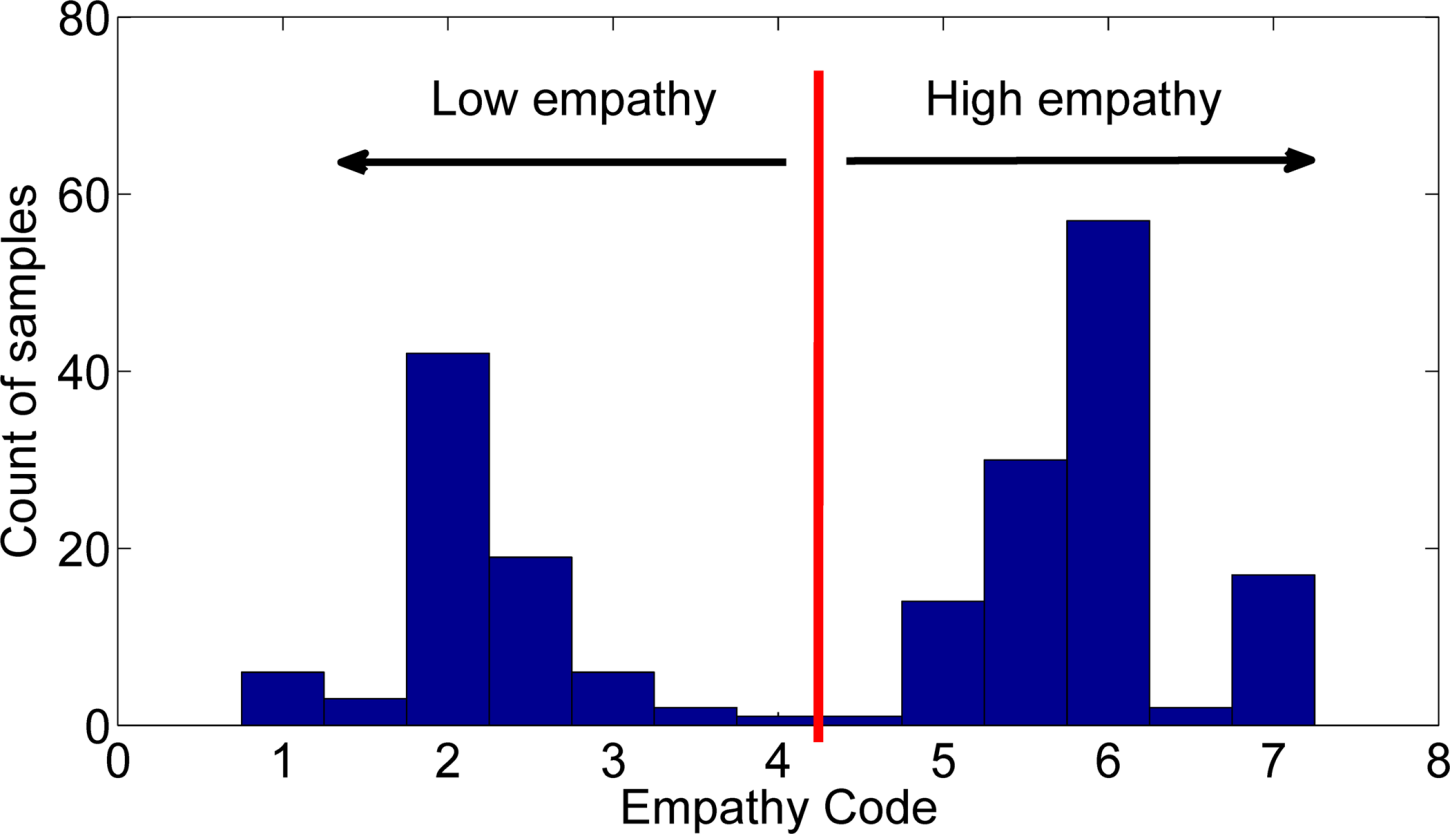
Distribution of human empathy ratings for the 200 sessions in the CTT trial.

[25] Each session in the CTT corpus was rated using the global indices from the MITI, which offers conceptually derived summary indices for measuring elements of MI skillfulness. The MITI 3.0 includes a global practitioner rating for empathy, scored on a 7-point Likert scale and intended to capture gestalt, “all at once” impressions of the session. A low empathy rating indicates that the counselor showed no interest in the client’s perspective, and a high rating indicates that the counselor demonstrated “a deep understanding of client’s point of view” (p.14). Raters received training and supervision in use of the MITI and were blind to assessment timing and practitioner identifiers throughout their scoring process. We used the averaged code value if a session was coded more than once. A histogram of the empathy ratings used in the study is shown in Fig 1. The inter-rater reliability of continuous empathy ratings was ICC = .60, which is in the range of previous studies. When we classified human empathy ratings into a binary high vs. low indicator inter-rater agreement was higher, Kappa = .74. High vs. low empathy classes have average empathy codes 5.90±0.58 vs. 2.16±0.55, respectively.

### Speech Signal Processing and Text-based Predictive Modeling Procedures

**Fig 2:**
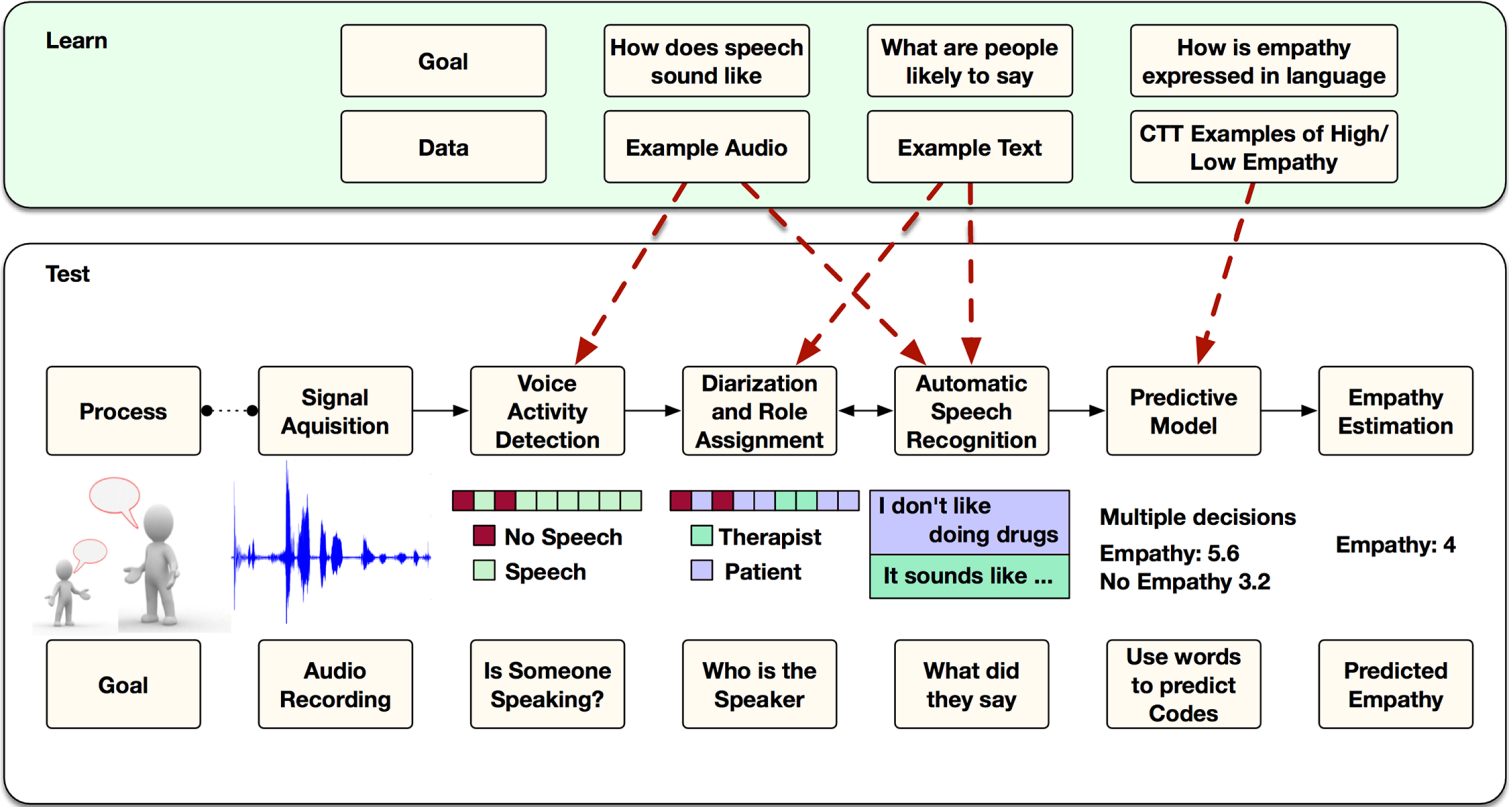
Overview of processing steps for moving from audio recording of session to predicted value of empathy. The lower portion of the figure represents the process for a single session recording, whereas the upper portion represents various speech signal processing tasks, learned from all available corpora (as indicated in the text).

The key steps and tasks required to move from an audio recording to a predicted empathy rating are presented in Fig 2 and described below. Please also refer to the technical supplement (S1 File) for more extensive details regarding system implementation.

#### Speech signal pre-processing

Starting from the audio recording of a session, several processing steps occur prior to automatic speech recognition. First, Voice Activity Detection (VAD) [26,27] is used to chunk the continuous, long audio stream into short segments, identified simply as either containing speech or not (i.e., silence or non-speech sounds). In this work, we employ the VAD system based on a variety of speech features and neural network models described in [28]. Average error time for the VAD on the CTT set is 12.6%.

Second, when multiple speakers are recorded together (i.e., without separate recording channels per speaker), it is necessary to identify the regions in the audio stream that belong to each unique speaker. This is important both for the predictive modeling of empathy, which is speaker dependent, but also assists the automatic speech recognition process as individual speakers have their own, unique acoustic characteristics. The process of identifying specific speakers in an audio recording is called diarization [18]. We employ the diarization systems based on agglomerative clustering in [29] and Riemannian manifold clustering [30] for the implementation. Average error time for the diarization on the CTT set is 18.1%. The diarization system simply identifies regions of the audio recording corresponding to each speaker (e.g., speaker 1 vs. speaker 2). As part of the language processing (below), it is possible to learn the types of words that are typical of a counselor vs. patient, which can then be used to identify the role of the speaker. For example, a patient tends to use more first person words such as ng to each speaker (e. a therapist tends to use more second person words such as s such as ng to each speaker (e.., speaker 1 vs. speaker 2). As part of the language processing (below), it is possible to learn the types of words that a”therapWe were able to automatically identify speaker roles for 75.5% of the CTT sessions, and for the rest we treated the entire decoded transcript as therapist language. For more algorithmic details please refer to the technical supplement.

#### Automatic Speech Recognition (ASR)

In addition to the utilization of a standard lexicon that maps words to pronunciation, the system iteratively learned the two statistical models employed by the ASR [19,21,31]: the Acoustic Model (AM) and Language Model (LM). The AM describes the acoustic properties of the phone combinations in the spoken language. The corpora of MI clinical trials used in this study have transcriptions with time-stamping for each talk turn, which we used to generate an MI-specific AM. Using an iterative optimization process, the AM converges to a set of statistical models describing every phoneme in terms of its acoustic properties.

The language model (LM) specifies the distribution of typical expected language usage to be decoded by the ASR. It characterizes language use by describing the probabilistic likelihoods of a single word or short, multi-word phrases, which are combined to get the likelihood of a word sequence in any length. These multi-word phrases (called n-grams) are useful in that they indicate the probability of speaking any word *w* at the present instant, given the preceding word or words *p*. Such probabilities are typically estimated as the count of occurrences of *w* following *p*, divided by the total occurrences of *p* in a large, representative text corpus [32]. In this work, the LM of the present ASR system was learned from both the MI transcripts as well as the general psychotherapy transcripts. We used the SRILM toolkit [33] in this implementation.

Once the AM and LM are in place, then the ASR system is ready to “decode” the audio into unique words for the CTT dataset. In the decoding step, the ASR system makes decisions based on the AM and LM jointly to derive the most probable word sequence given the audio recording. In this work, we employed the widely accepted Kaldi toolkit for the implementation of the ASR system including training the AM and decoding the speech utterances [34]. An online system (i.e. decoding the text as audio recording comes in, instead of waiting for the completion of the treatment) based on the Barista framework is in development and publicly available [35]. The accuracy of ASR was evaluated by examining the Word Error Rate (WER) in the CTT dataset, which is the percentage of the total words inaccurately decoded compared to human transcriptions, including word substitution, insertion, and deletion.

#### Building a text-based predictive model of empathy

The predictive model for empathy was trained using human generated transcripts and resulted in a scoring algorithm that given input words, outputs an empathy score. The present analyses examined two separate models, including one in which empathy was treated as a binary outcome with high vs. low empathy sessions, and a second model that treated empathy as a dimensional construct. Both approaches led to scoring algorithms that were then used to generate either empathy classifications (high vs. low) or empathy scores in new sessions that the model had not seen, using text inputs automatically generated by the ASR system. It was developed in two stages.

First, high vs. low empathy language models were estimated, which provided the likelihoods of a given utterance being generated by each of the above models. The quotient of high-empathy likelihood divided by the sum of both high and low empathy likelihoods was mapped into an utterance-wise empathy score that ranged from 0 to 1. A session level empathy score, also between 0 and 1, was determined by taking the mean of all utterance scores for the counselor. To create an overall empathy score, a regression function is needed to jointly map the empathy scores based on either words or short phrases. For this mapping, we used linear regression and uni-gram (a single word without context), bi-gram (a word given the previous word), and tri-gram (a word given the previous two words) language models of high vs. low empathy. Likewise, a decision function is needed to jointly map the scores to an overall binary classification of high vs. low empathy. This decision function was derived using a machine learning method called Support Vector Machine [26], which automatically derives an optimal dividing threshold such that training samples from the two classes are furthest to the dividing threshold (i.e., maximizing the margin from any training sample to the threshold). We use the LIBSVM toolkit [36] for its implementation.

Importantly, this entire process was evaluated using cross-validation. The ultimate goal is to predict empathy in new sessions that have not been manually transcribed and were not included in the empathy model itself. To simulate this goal, a leave-one-counselor-out cross-validation (CV) was used to estimate the accuracy of the predictive model. [37] Here the empathy prediction model was trained on all but one counselor’s sessions and then the model predicted the empathy of the one, left-out counselor’s sessions based on the words generated by the ASR transcripts for those sessions, meaning that predictions of empathy are fully automatic and independent to the counselor. The CV procedure then iterates over all counselors.

We used the average of human-annotated empathy codes as a gold standard reference for comparison (i.e., if a session was rated by two different raters, the gold standard was the average of those ratings). Empathy prediction was evaluated in several ways, 1) the Pearson’s correlation between model predicted empathy scores and human annotated empathy codes, 2) overall accuracy of classification of high empathy codes (total proportion of empathy codes correctly classified), as well as 3) recall (sensitivity; number of true high empathy codes divided by the number of actual number of high empathy codes), 4) precision (specificity; number of true high empathy codes divided by total number of high empathy codes generated by the model), and 5) F-score. Finally, because human-based empathy codes contain measurement error themselves, we report the agreement statistics of human coders. The inter-rater reliability statistics provide the true upper-bound for the current predictive task, as model-based comparisons could never exceed this value.

As a sensitivity check of how the ASR procedure affects the accuracy of empathy modeling, we also examined prediction of empathy codes derived by manual transcription, and the ASR decoded transcription using human-based diarization. These comparisons provide an upper bound on the prediction of empathy codes from text if both ASR and diarization were highly accurate.

## Results

In Table 2 we compare the performance of empathy predictions. In the first row we show the “chance” prediction, i.e. guessing high empathy for all sessions. This yields 60.5% accuracy, 100% recall, 60.5% precision, and 75.4% F-score (a weighted average of recall and precision) for detecting high empathy, due to imbalance in high and low empathy sessions in the data. An estimate (see Table 2) of human coder agreement found 89.9% accuracy, 87.7% recall, 93.7% precision, and 90.3% F-score comparing individual opinions to the gold standard, shown in the second row.

**Table 2:** Empathy prediction performance.

Model	Correlation	Accuracy (%)	Recall (%)	Precision (%)	F-Score (%)
Chance level	-	60.5	100.0	60.5	75.4
Coder (individual vs. average) agreement ^a^	-	89.9	87.7	93.7	90.3
Human Transcription	0.71	85.0	96.7	81.8	88.6
Human Diarization	0.65	80.5	93.4	78.5	85.3
Full VAD, Diarization, ASR^b^	0.65	82.0	91.7	81.0	86.1

^a^ These results were calculated on 63 sessions instead of 200. On sessions coded by multiple coders, the average opinion is used to derive the dichotomous (binarized) decision. The opinion of the individual coder is compared with the average decision to establish this coder-agreement.

^b^ Result is fully automatic, no human intervention in algorithm.

In the third row, we show the results when the empathy prediction model employs manually (e.g., human) generated transcripts and the manually labeled speaker role and timing. This yielded a correlation of 0.71 with the gold standard ratings in the raw range. In terms of binary classification, this setting achieved 85% accuracy, 96.7% recall, 81.8% precision, and 88.6% F-score for detecting high empathy sessions. These results closely approached human performance.

In the fourth row, we show the results when the empathy prediction model employs manually labeled speaker role and timing information, but automatically generated transcripts through ASR. The ASR had a mean WER of 43.1%. This setting achieved a correlation of 0.65 to the gold standard, and 80.5% accuracy, 93.4% recall, 78.5% precision, and 85.3% F-score for detecting high empathy sessions.

In the last row, we show the results achieved by the fully automatic system, including VAD, diarization, ASR, and empathy prediction. The ASR had a negligible increase in mean WER to 44.6%. Given extreme variation in recording quality, the range in WER was high (19.3% to 91.6%). In real patient sessions where recording conditions were even less controlled, WER was strongly related to signal-to-noise-ratio—a signal quality metric—in the audio file (*r* = .5). There was no significant difference in ASR performance with respect to ethnicity, gender, or age of the therapist. The empathy prediction had a correlation of 0.65 to the gold standard ratings generated by humans. In terms of binary classification, the system yielded 82.0% accuracy, 91.7% recall, 81.0% precision, and 86.1% F-score. These are slightly higher than the case in the previous row, and close to the one with manual transcripts.

We report more details about the performances of the speech processing components in the technical supplement.

## Discussion

The current study represents the first demonstration that it is possible to record a psychotherapy session and automatically evaluate the quality of one of its core components–in this case, therapist empathy. The automatic classification of high and low empathy sessions extends a literature demonstrating that linguistic inputs from human transcriptions can successfully measure expert-derived quality metrics of psychotherapy. This includes text-based classification of specific therapist behaviors in MI [14], behavioral vs. integrative couples therapy [38], and interventions from cognitive behavioral and psychodynamic sessions. [15] In addition, these findings compliment previous research demonstrating the possibility of using vocal prosodic features to detect patient distress in PTSD (post traumatic stress disorder) [39], depression [40], and therapist empathy. [41–42] Moreover, recent research suggests that text-based features (i.e., language measures) may be more effective than using vocal prosodic features alone when classifying high and low empathic sessions [41]. While [41] employed manually annotated speaking time and speaker information, this work integrates various speech processing modules such that the end-to-end system could be made fully automatic, potentially providing better prediction of human ratings than the results presented in this paper. The software toolkit of the system is available at *https*:*//github*.*com/usc-sail/barista* and is under on-going updates.

As an illustration, we provide examples of high vs. low empathy language in Table 3. We list the highest ranked tri-grams in terms of their discriminative power for high vs. low empathy, which is computed based on the difference of their likelihoods when evaluated against the high vs. low empathy language models times their frequency in the corpus. Note that these tri-grams are *not* the cues directly used for empathy prediction but offer an illustration of language patterns that were useful in algorithmically discriminating high and low empathy sessions. It appears that high empathy words tend to suggest reflective listening with expressions such as “sounds like”; while low empathy words relate to giving instructions or probing information.

**Table 3:** High vs. low empathy tri-grams.

High empathy		Low empathy	
it sounds like	a lot of	during the past	please answer the
do you think	you think about	using card a	you need to
you think you	you think that	past twelve months	clean and sober
sounds like you	a little bit	do you have	have you ever
that sounds like	brought you here	some of the	to help you
sounds like it’s	sounds like you’re	little bit about	mm hmm so
p s is	you’ve got a	the past ninety	in your life
what I’m hearing	and I think	first of all	next questions using
one of the	if you were	you know what	you have to
so you feel	it would be	the past twelve	school or training

Results in this work have potential implications for the development of therapist expertise, the implementation of evidence-based treatments, and mechanism research in psychotherapy. Specific, immediate feedback is critical to the development of expertise across a broad swath of domains and skills. [43] However, specific, immediate feedback is precisely what is missing from the practice of psychotherapy. [44] As a result, many have argued that “expertise” in psychotherapy is challenging to attain. [45] Clinical supervision in the community–when available–is typically based on verbal reports from therapists to their supervisors. Yet, supervisees may not disclose details of cases when they are performing less competently, [46] and there may be little correlation between provider report and their actual behavior with patients. [47]

Carroll [12] recently noted that a “…major barrier to implementation of virtually all empirically validated therapies is the lack of quality-control measures in most mental health and addiction-treatment systems” (p. 10). Essentially, providers may receive training in any number of specific interventions, but without ongoing feedback and supervision, it is unlikely [10] that they will continue to maintain high levels of treatment fidelity. Evaluation of interventions like MI that do not require humans as the sole method of assessment may ultimately allow feedback proximal to the clinical encounter, and the scalable evaluation of disseminated interventions in the community in a way that was previously thought to be infeasible.

Finally, computational evaluation of specific components of psychotherapy offers the potential to alter the scope of mechanism research. A recent meta-analysis of MI mechanism research consisted of only 783 sessions, [48] which is less than the total number of sessions in large, single clinical trials. Beyond MI, many well conducted large-scale trials that compare psychotherapies with different theoretical underpinnings are only able to investigate the content of a very small percentage of total sessions (e.g., psychodynamic vs. cognitive behavioral therapy [49] which coded ½ of 1% of sessions). Here the labor intensiveness of behavioral coding limits a thorough evaluation of internal validity (did therapists do what they were trained to do, and not what they weren’t supposed to do), but also limits more detailed investigations of how treatments work. Tools that can be trained to detect specific indicators of provider fidelity based on a small number of sessions could then be used to evaluate thousands of sessions, improving the specificity clinical trials in psychotherapy and the scale of mechanism research.

### Limitations and Future Directions

A primary limitation of the current study is the relatively limited sample size used for designing the ASR. The ASR system employed state-of-the-art techniques, but the performance was notably worse than those on standard test beds (e.g. 15% WER for transcribing broadcast news). Several factors contribute to the increased WER, including relatively small training data size, spontaneous rather than read speech, overlapped speech in interaction, varied recording conditions often with notable ambient noise and distortion, and far field speech recordings. For example, in the RP sessions where the recording conditions were less controlled, there was a -0.5 correlation between WER and SNR (signal-to-noise ratio, an audio quality measure). However, the end goal of this work is to predict empathy. The accuracy of ASR is only an intermediate result. Despite sub-optimal ASR performance, classification accuracy was quite high, approaching human reliability. This suggests that improvements in audio quality may lead to modest improvements in performance, but that even with relatively high word error rates, there was sufficient linguistic signal captured by the statistical n-gram language models to discover differences in therapist empathy that were detected by human raters. Future research that utilizes computational tools to assess clinical interactions should take care to capture audio in a fashion that will facilitate high quality recordings (e.g., quality microphones, separate channels for each speaker). Moreover, we have verified that there is no variability in ASR performance with respect to ethnicity, gender, or age of the therapist. Though speech properties vary for different speaker groups, an ASR trained with a mixture of diverse training data should be robust to such heterogeneities.

In addition, the prediction models utilized only linguistic content–essentially provider words, whereas there is evidence that the expression of empathy also involves acoustic channels–how a word is said besides what is said. Moreover, simple turn-taking cues such as the number of talk turns of therapist may correlate with empathy. [50]

There are a variety of future directions for the present work. At the broadest level, this work can be extended to additional quality indicators beyond empathy, both within MI and in other treatment approaches. The necessary predictive models can be extended to include both prosodic (i.e., acoustic) and linguistic (i.e., text) inputs to capture both what and how communication occurs in session. As predictive models for provider fidelity become more accurate, this should clarify what is not coded by these instruments, assisting the search for new mechanisms of change in psychotherapy.

Furthermore, the current system includes several signal processing steps, and employs statistical n-gram language models and SVMs, making it challenging for a human to directly track the cause of errors in session-level empathy prediction. We plan to analyze the errors in-depth and improve system robustness when the system is deployed in real clinical settings where more samples and human judgments are accessible. Human expert’s timely feedback and correction towards session level prediction may improve the effectiveness of the system. This may be achieved by an “active learning” approach [51], which is a machine learning technique that automatically selects the optimal subset of samples requiring human supervision, so that the cost of human labor is reduced in order to update the models in the system.

Finally, the current system is a prototype model. At the moment, the development of computer based tools for “automatically” evaluating specific processes in psychotherapy remains an area of active research, combining expertise from engineering, psychology, linguistics and computer science. This work is expensive and labor intensive. However, the “labor” is different than that typically employed in a psychotherapy study where a group of coders is trained to evaluate a set of studies and then is disbanded–a process that is repeated in many labs around the world, and never gets any faster, and ultimately has the same rate-limiting factor, humans. While the work we are pursuing starts with human input, humans are not the rate-limiting factor, only processing speed of computers, and the ongoing improvement of various ASR and natural language processing techniques, and machine learning algorithms. With time and additional research, we hope and expect that these methods may become commonly utilized tools in psychotherapy research and real-world applications to enhance the effectiveness of training and delivery of services.

It is also important to acknowledge that there are important ethical considerations during the process of developing an “expert system” that is trained by human ratings, but where the human is no longer the primary evaluator. For example, what are therapists and administrators to do when they feel the system makes errors (e.g., rating a session as low empathy, when an expert human rater believes it to be a high empathy sessions). First, the ethical implications of algorithmic evaluation must be viewed in the context of the current standard of care. At present, counselors primarily exist in a vacuum–essentially no evaluation at all is the current standard–there is no scalable mechanism for evaluating the quality of psychotherapy in the community [44]. This clinical reality results in a lack of accountability to any professional standard and introduces other ethical concerns that an expert system such as the one we have described here might ultimately begin to address. Second, human evaluation of psychotherapy is far from perfect and suffers from ongoing reliability problems both within and between coders [24], thus human rating of a given therapist is certainly not above reproach. Finally, we do not envision a feedback system based on this algorithm to be implemented in the absence of ongoing human supervision–i.e., humans should remain in the loop. In particular, computationally derived feedback should not be used in a punitive fashion, but in order to support ongoing provider self-evaluation, as well as clinical trainings that often do not involve ongoing feedback, and as an adjunct to human based clinical supervision when it is available. Certainly in early system implementations that require ongoing research to validate system accuracy, we recommend that the system be used as an adjunct to typical evaluation protocols that can be used for therapist benefit. As systems like this one proliferate in number and accuracy improves, it is likely that they will be utilized as one component of an overall evaluation of provider performance. Thus, research on how accurate these systems are and how they can best be used to support providers and improve the quality of mental health services remains critical.

Despite important limitations that are necessarily involved in the development of this initial system, we feel that the results we describe here provide highly encouraging evidence that automatic systems are possible. As these systems become more robust given more training data, and the computation platforms become more accessible (e.g. on smart phone or tablet), clinical applications will be possible, allowing near real-time feedback to therapists, and the incorporation of user feedback during training. It is our hope that such tools may be one additional tool that could increase the provision of high-quality treatment in mental health.

#### Conclusions

At present, clinical practice and research in substance abuse treatment and mental health generally involves little to no direct evaluation of provider behavior. Psychotherapy research has long relied on the labor intensive and error-prone process of collecting observer ratings. However, real-world training and service delivery demands are orders of magnitude larger than even our largest research studies. In many locales, the service delivery system and counselors are in place, but the training and quality assurance methodology (i.e., behavioral coding) are hopelessly mismatched. The utilization of computational tools from speech and language processing present a technological solution that may ultimately scale up training, supervision, and quality assurance to match the service delivery need.

## Supporting Information

S1 File S1_File.pdf. (PDF)
