# Supplementary material for: "Rate My Therapist": Automated Detection of Empathy in Drug and Alcohol Counseling via Speech and Language Processing
Source: PLoS One. 2015 Dec 2;10(12):e0143055. doi: 10.1371/journal.pone.0143055 (PMC4668058; doi:10.1371/journal.pone.0143055)
Supplement: S1 File — (PDF) [file pone.0143055.s001.pdf]

Supplement for "Rate my therapist": Automated detection of empathy in drug and alcohol counseling via speech and language processing

Bo Xiao*, Zac E. Imel, Panayiotis G. Georgiou, David C. Atkins, Shrikanth S. Narayanan

October 7, 2015

1 Speech Processing and Automatic Speech Recognition

In this section we introduce the technical details about the speech signal processing components in the proposed empathy prediction system, including *Voice Activity Detection* (VAD), Diarization, *Automatic Speech Recognition* (ASR), and speaker role matching.

1.1 Voice Activity Detection

Voice Activity Detection (VAD) aims to separate regions of the audio signal that contain speech from those that do not contain speech (*e.g.,* silences and environmental noises). The present research employed the VAD module developed by Van Segbroeck *et al.* [1]. The module uses a variety of spectral and acoustic features extracted from the audio signal, including: (I) spectral shape, (II) spectro-temporal modulations, (III) periodicity structure due to the presence of pitch harmonics, and (IV) the long-term spectral variability profile. After extracting the raw features, we normalize each feature dimension by the variance.

Using *training* data that are already manually annotated into speech and non-speech regions, we train a neural network model for the VAD task. The parameters of the VAD system are optimized on a separate set of audio signals referred to as the *development* set. Both the training and development data are independent of the data used for the evaluation of our system (*i.e.,* predicting empathy codes) described in the body of this work. The two sets employ a sample of 67 sessions drawn from the MI randomized trials, totaling 5.2 hours and 2.6 hours in length, respectively.

The output of the VAD model is in a form of voicing probabilities from 0 to 1. We transform this to a segmentation format as follows: Initially, we set a threshold of 0.5 in voicing probability to convert the continuous probabilities into 0 or 1 speech labels. To eliminate long speech segments that are difficult to handle in later steps, and are not likely to happen in dyad, we adaptively increase the initial threshold of 0.5 until all speech segments are below a maximum length of 60 seconds. Moreover, very short breaks of speech segments may not be necessary, and could break the continuity of utterances. We merge two consecutive speech segments if the gap is below 0.1 seconds and the combined segment length is still below 60 seconds. Finally, we drop stand-alone short segments, which are less than 1 second and likely to be noise in speech detection. This gives the initial VAD output. For further details on the VAD models, please see [1].

*Corresponding author for Supplemental Material: boxiao@usc.edu

1.2 Diarization

The corpora used in the current work were collected using a single, far-field mic, and hence, the therapist and patient voices are contained in a single recording and audio file. The diarization module aims to separate the speech of the therapist from the patient. Since it is based on acoustics only, it can separate the two speakers, but it is unable to assign their roles (*i.e.,* it can separate speakers but not say who is therapist and who is patient), which is handled in a separate step. We can employ diarization information to (1) enable the ASR module to adapt the acoustic models in an unsupervised fashion to the speaker patterns thus improving transcription accuracy, (2) identify what each speaker said after the ASR module, and (3) in coordination with the ASR transcript identify the role of each speaker.

Given the application scenario, we assume the number of speakers in the audio recording is known as two speakers — the therapist and the patient. Thus the diarization mainly includes two steps: segmenting speech into speaker homogeneous segments, and clustering these segments by assigning a speaker label (speaker #1 or speaker #2) to each one.

We consider two diarization methods, and run the processing for two iterations, described as follows.

1. We employ the method in [2], which takes the VAD results and Mel-Frequency Cepstrum Coefficients (MFCC) as inputs, segments the speakers by Generalized Likelihood Ratio (GLR), and clusters the speakers by agglomerative clustering (results denoted as D-1). In parallel, we employ the method in [3], which takes the same input and GLR speaker segmentation approach, but uses Riemannian manifold method for speaker clustering (results denoted as D-2).

2. Apply the ASR based on the diarization result of the latter approach (D-2) and obtain the time alignment information from the best decoding path. Extract speech *vs.* non-speech timing information from the alignment and regard that as a new type of VAD, while disregarding the decoded text.

3. Employ the ASR derived VAD information and the MFCC features, run the method in [3] again, with the setting of slicing speech to a finer degree of 1 minute long segments, which is shown in [3] to improve the accuracy (diarization results denoted as D-3).

The diarization approaches employed in this work do not have a pre-trained model, but learn from the data in an unsupervised manner. We use a rule-based fusion process to exclude erroneous results in D-3 while trying to recover from D-2 and D-1. We employ the following session-level features for the fusion: (I) percentage of speaking time by each speaker; (II) longest duration of a single speaker's turn. Our rules are based on the intuitive assumption that it is unlikely in counseling that one speaker keeps speaking for very long time. Thus, we define outliers as session-level features that are three times the standard deviation away from the mean value. The final fusion of diarization results based on these rules is as follows.

4. If D-3 is not an outlier, we take D-3 as the final result; otherwise, if D-2 or D-1 is not an outlier, we take them in turn as the final result. If both D-2 and D-1 are outliers as well, we take D-3 as the final result.

1.3 Automatic Speech Recognition (ASR)

For the purposes of the present work, we designed an *Automatic Speech Recognition* (ASR) system that incorporates a large vocabulary and is able to recognize continuous speech. The system is

implemented using the Kaldi library [4] for both training and testing purposes. For the future clinical deployment and real-time feedback, we are currently developing an online (*i.e.,* real time) version of the system using the Barista framework [5]. In the following we describe details of the system in various aspects.

Feature We transform input audio recordings to 16kHz sampled, single channel waveform. We then extract standard 13-dim MFCC features from the signal and append the first and second order time differentials of the MFCC features to the complete feature vector.

Dictionary We employ the combined lexicon from the WSJ [6] and the Switchboard [7] corpora. These dictionaries do not fully cover our domain. To improve coverage for the domain specific words, we manually added pronunciations to the dictionary for the words that appeared over 8 times in the training data. For example, *vicodin* (a drug) and *mm* (a filler word) were added to the combined dictionary. We ignored words from our dataset that were of low frequency (appearing less than 8 times) as they are mostly misspelled (*e.g.,* "quesitons", "uglly", "somwhehere", *etc.*) or made-up words (*e.g.,* "twelvish", "prereqs", "worriness") due to errors in transcription and the oral conversation scenario. In total there were only 322 word tokens ignored, less than 3/10000 of all word tokens in the training data.

Text The manually derived transcripts need normalization to regulate the text format for training the ASR. In the transcripts, overlapped speech regions are marked by a "<...|...>" format, where the words before and after the bar "|" belong to the primary and secondary speakers, respectively. In total there are 15895 instances of overlapped speech, compared to 36907 talk turns (multiple overlaps may happen in one turn). However, since ASR is not able to decode overlapped speech, we keep only the longer utterance in overlapped regions. We keep all the repetitions and fillers as they are in the transcript. We normalize non-verbal vocalization annotations into two types including "[laughter]" and "[noise]". Finally we replace underscores by spaces, and remove punctuations and all special characters.

AM We train the *Acoustic Model* (AM) for several iterations. First, we train a GMM-HMM based AM on short utterances with a mono-phone setting. This initial model is gradually expanded to a tri-phone structure fitted to the entire training set. We then employ feature Maximum Likelihood Linear Regression (fMLLR) and Speaker Adaptive Training (SAT) techniques to improve the model. Finally, we train a Deep Neural Network (DNN) based AM following the previous model.

LM A *Language Model* (LM), representing the probabilistic occurrence of sequences of words, is critical for the accurate performance of the ASR. We train two tri-gram (*i.e.,* 3-gram) LMs using Kneser-Ney smoothing [8]. The first is a background model, trained on a large in-domain text corpus of "General Psychotherapy" interactions (see description in [9]). With more training data, this LM is better able to represent the probabilistic occurrence of language usage, but it is of a more general nature and less matched to the interactions at hand (*i.e.,* the language in general psychotherapy is far more variable than that found within a corpus of MI only). The second LM is trained on the MI randomized trials. Although the data is sparse, the LM is able to better capture the specific language usage in MI since it better fits the type of conversation compared to the background model. The two LMs are mixed together, with a mixing weight optimized on a small sample held-out from the MI randomized trials corpus. This produces the final LM for the ASR. For this process we employ the SRILM toolkit [10].

We compare the performance in two settings: having manually labeled speaker boundaries and speaker roles, or totally automatic using the signal derived diarization results. For the latter, we employ the ASR twice. The additional first-pass ASR provides a rough transcript employed in the 3rd step of the diarization process as described above.

1.4 Speaker role matching

The diarization module only separates distinct speakers but does not associate speakers with their roles in the interaction (*i.e.,* which speaker is the therapist and which is the patient). In order to model therapist language, we need to match the speakers with their roles automatically, based on their specific language styles. For example, the therapist may ask more questions and use the word "you" more often. The degree by which each speaker's language matches language generated by other speakers of the same role can be employed to identify the role of the speaker. The detailed procedure is listed as follows.

1. Train therapist (T) and patient (P) specific language models, based on the labeled transcripts of therapist and patient language in the training corpus (MI randomized trials). These two models represent the speaking style of the two participants based on their roles. We train those, as described above, as tri-gram LMs with Kneser-Ney smoothing, using SRILM.

2. For vocabulary consistence and robustness, mix the final ASR LM into each role-specific LM with a small weight.

3. Let the speakers be S-1 and S-2. Compute the perplexities of the decoded utterances by S-1 and S-2 on the two role-specific LMs, respectively. Denote $\mathrm{ppl}_{1,T}$, $\mathrm{ppl}_{1,P}$ as the perplexities of S-1 on therapist and patient LMs, respectively; similarly obtain $\mathrm{ppl}_{2,T}$ and $\mathrm{ppl}_{2,P}$ by S-2.

$$\mathrm{ppl}(w_1 \cdots w_m) = (P(w_1 \cdots w_m))^{-\frac{1}{m}} = e^{-\frac{L(w_1 \cdots w_m)}{m}} \tag{1}$$

Here perplexity is defined as a metric based on normalized log-probability of the text, shown in (1). $w_1 \cdots w_m$ represents the word sequence of length m. $P(w_1 \cdots w_m)$ and $L(w_1 \cdots w_m)$ stand for the likelihood and log-likelihood of the word sequence, respectively. A smaller perplexity is associated with a higher likelihood, suggesting a better fit of the text to the LM.

$$\mathrm{ppl}_{1,T} \leq \mathrm{ppl}_{1,P} \quad \& \quad \mathrm{ppl}_{2,P} \leq \mathrm{ppl}_{2,T} \tag{2}$$

$$\mathrm{ppl}_{1,P} < \mathrm{ppl}_{1,T} \quad \& \quad \mathrm{ppl}_{2,T} < \mathrm{ppl}_{2,P} \tag{3}$$

4. We compare the perplexity results as follows.
If (2) holds, we match S-1 to therapist and S-2 to patient, because S-1 has the smaller perplexity on the therapist LM, and similarly S-2 has smaller perplexity on the patient LM.
If (3) holds, we match S-1 to patient and S-2 to therapist.
If neither of (2) or (3) holds, we take both S-1 and S-2 as the therapist. We tend to incorporate more utterances into therapist language. This compromises the transcript purity but ensures that anything the therapist may have said is included in the transcript since our target is to model the therapist's empathy behavior.

5. Finally, we check if the diarization result is highly biased, *i.e.,* if one speaker occurs more than 10 times of the other speaker. In such cases the perplexity comparison might not

be effective due to the sparsity of text input. The biased distribution may be due to an erroneous diarization that may have clustered speech from both speakers against nonverbal vocalizations. As a solution, we match the speaker assigned more utterances with the therapist to ensure more complete coverage of therapist language.

2 Language Modeling for Empathy Prediction

2.1 Maximum Likelihood based N-gram Language Model

We train high *vs.* low empathy N-gram LMs based on the manual transcripts of high *vs.* low empathy sessions in the CTT dataset, respectively. Mathematically, N-gram models are usually constructed by estimating the conditional word probabilities following the Maximum Likelihood criteria. Such an estimation is often implemented by a count-and-divide approach for the initial step and refined by various smoothing techniques to improve the robustness against data sparsity [8]. Specifically, the above LMs are again tri-gram LMs with Kneser-Ney smoothing, implemented with SRILM. For robustness we again mix the final LM in ASR (that provides good language coverage) to high and low empathy LMs with a small weight of 0.1.

For an utterance u containing a word sequence $w_1,\ w_2,\ \ldots,\ w_m$, the LM estimates the likelihood of u being generated by the specified language model. A tri-gram model makes an assumption that a word occurrence depends only on the previous two words occurring. For example, the probability of generating utterance u by a LM is given by $P(u)$, shown in (4). Following the rule of conditional probability, $P(u)$ is expanded to the form in (5) and (6). With the tri-gram assumption, further dependencies are dropped so that $P(u)$ becomes the form in (7). It is possible in practice to estimate statistically robust $P(w_i|w_{i-1}w_{i-2})$ from relatively large size text data, which can be used to derive the likelihood $P(u)$ of the entire utterance. Likewise, a bi-gram (*i.e.,* 2-gram) model assumes a word only depends on the previous one word; and a uni-gram (*i.e.,* 1-gram) model assumes all words are independent.

$$
\begin{aligned}
P(u) &= P(w_1 w_2 \cdots w_m) && (4)\\
&= P(w_1)P(w_2|w_1)P(w_3 w_4 \cdots w_m|w_2 w_1) && (5)\\
&= P(w_1)P(w_2|w_1)\prod_{i=3}^{m} P(w_i|w_{i-1}w_{i-2}\cdots w_1) && (6)\\
&= P(w_1)P(w_2|w_1)\prod_{i=3}^{m} P(w_i|w_{i-1}w_{i-2}) && (7)
\end{aligned}
$$

We denote the likelihoods of generating u by the high *vs.* low empathy LMs as $P(u|H)$ *vs.* $P(u|L)$, respectively. Following Bayes' rule, we model the posterior probability $P(H|u)$ by the likelihoods as in (8), where we assume equal prior probabilities $P(H) = P(L)$.

$$
P(H|u) = \frac{P(u|H)P(H)}{P(u|H)P(H) + P(u|L)P(L)} = \frac{P(u|H)}{P(u|H) + P(u|L)} \tag{8}
$$

We compute a session level empathy score α_n as the average of utterance-level evidences as shown in (9), where $\mathbf{U}$ is the set of therapist utterances, n is the order of the n-gram LM, and $P_n(H|u)$ is the posterior based on the n-gram LM.

$$\alpha_n(\mathbf{U}) = \frac{1}{K}\sum_{i=1}^{K} P_n(H|u_i), \quad \mathbf{U} = \{u_1, u_2, \ldots, u_K\}. \tag{9}$$

Finally, due to data sparsity, we carry out the above analysis in a leave-one-therapist-out cross-validation. That means we keep one therapist's sessions out from the training, and train the high *vs.* low empathy models on all other sessions in the CTT set. Once the models are obtained, we test the left out sessions so as to predict the empathy codes. We then repeat this process for all therapists. The final report of performance is based on the overall results of all iterations of the cross-validation. For the 200 sessions in the CTT set, there are 133 therapists, so that we repeat the LM training and testing for 133 times. In this way the prediction of empathy is always independent to the therapist.

2.2 Fusion of Empathy Scores for Code Prediction

Given the methods just described, we have obtained empathy prediction in the form of averaged posterior probabilities α_n, $n = 1, 2, 3$. These cues may provide complementary information about empathy. Therefore, we propose a fusion step using Linear Regression to integrate them into a single predictor. Here we take the annotated MITI empathy code as dependent variable, and employ α_n as independent variables. Meanwhile, we predict the class of high *vs.* low empathy, using linear Support Vector Machine (SVM) [11] implemented in the LIBSVM toolkit [12]. Here we take the label of high or low empathy class as a binary target variable, and take the 3-dimensional scores of α_n as features.

The above analyses are under a leave-one-therapist-out cross-validation (*i.e.,* ($N - 1$ training) *vs.* (1 evaluation), for N times), where N is the number of distinct therapists. However, in order to train the regression and SVM models, we need an adequate number of samples being tested by the empathy prediction methods, but a single therapist has too few sessions to start training the linear regression or SVM model. As a solution, we conduct an internal leave-one-therapist-out cross-validation in each cross-validation round, within the training part of the data (*i.e.,* [($N - 2$ **empathy model training) *vs.* (1 empathy model testing / SVM training when the** $N - 1$ **iterations are finished)**] *vs.* (1 evaluation), for a total of $(N - 1) \times N$ times). Such a scheme allows training the regression and SVM models on the empathy scores in the internal cross-validation ($(N - 1)\times$ term) , which are then tested on the empathy scores of the left out therapist in the main cross-validation ($\times N$ term).

2.3 Data usage summary

As noted in the main text, three different corpora were used in the present models. We present a summary of the data corpora usage in our work, as shown in Table 1.

3 Supplemental Results of Speech Processing

In this section we provide some additional results of the speech processing components. In Table 2 we list session-wise average false alarm (detecting non-speech as speech), miss (detecting speech as non-speech), and total error rate for the initial VAD module. We also list average false alarm (non-speech marked as speech), miss (speech marked as non-speech), speaker error rate (wrong speaker label), and total error rate for the final diarization results. In the implementation, we evaluate the VAD and diarization performances against manual annotations on speaking-turn level. The

Table 1: Summary of data corpora usage

Corpus	Phase	VAD	Diarization	ASR-AM	ASR-LM	Role	Empathy
MI randomized trials	Train	✓		✓	✓	✓	
	Test						
General Psychotherapy	Train				✓	✓	
	Test						
CTT	Train						✓
	Test	✓	✓	✓	✓	✓	✓

timing marks ignored gaps, backchannels, and overlapped regions within turns. As a result there are inherent errors in the reference data. However, these errors should not affect the conclusions significantly, since their offset-times are small.

Table 2: VAD and diarization performance.

Results	F.A. (%)	Miss (%)	Spk. err. (%)	Tot. err. (%)
VAD	5.8	6.8	-	12.6
Diarization	4.2	6.7	7.3	18.1

In Table 3 we report session-wise average ASR performance in terms of substitution (replacing a word with other word or words), deletion (missing a word or words), insertion (adding a word or words), and total Word Error Rate (WER)for the cases of decoding with manual or automatic diarization. The reference transcripts are generated by human annotators, and the speech recognition is based on the automated processes described above (ASR). The diarization however is done in two different methods: through human annotations (manual; the human decides who is speaking as indicated in the transcript) or through an automated machine process (automatic diarization). Thus we have two specific error rates for ASR, one when diaraization is done by humans (manually via the transcript), and the other automatically.

We see that in the automatic diarization case there is a slight increase in WER, which might be a result of VAD and diarization errors, as well as the influence on speaker adaptation effectiveness. For the fully automatic case, 151 sessions (75.5%) found a match of speaker roles, while 49 sessions failed to find a match.

Table 3: ASR performance for manual and automatic diarization cases.

Condition	Sub. (%)	Del. (%)	Ins. (%)	WER (%)
Manual diarization	27.1	11.5	4.6	43.1
Automatic diarization	27.9	12.2	4.5	44.6

References

[1] M. Van Segbroeck, A. Tsiartas, S. S. Narayanan, A robust frontend for VAD: Exploiting contextual, discriminative and spectral cues of human voice, in: Proc. InterSpeech, 2013, pp. 704–708.

[2] W. Wang, P. Lu, Y. Yan, An improved hierarchical speaker clustering, ACTA ACUSTICA 33 (1) (2008) 9.

[3] C. W. Huang, B. Xiao, P. Georgiou, S. Narayanan, Unsupervised speaker diarization using riemannian manifold clustering, in: Proc. Interspeech, 2014, pp. 567–571.

[4] D. Povey, A. Ghoshal, G. Boulianne, L. Burget, O. Glembek, N. Goel, M. Hannemann, P. Motlicek, Y. Qian, P. Schwarz, J. Silovsky, G. Stemmer, K. Vesely, The kaldi speech recognition toolkit, in: Proc. ASRU, 2011.

[5] D. Can, J. Gibson, C. Vaz, P. Georgiou, S. Narayanan, Barista: A Framework for Concurrent Speech Processing by USC-SAIL, in: Proc. ICASSP, 2014, pp. 3306–3310.

[6] D. B. Paul, J. M. Baker, The design for the wall street journal-based csr corpus, in: Proceedings of the workshop on Speech and Natural Language, Association for Computational Linguistics, 1992, pp. 357–362.

[7] J. J. Godfrey, E. C. Holliman, J. McDaniel, SWITCHBOARD: Telephone speech corpus for research and development, in: IEEE International Conference on Acoustics, Speech, and Signal Processing, Vol. 1, IEEE, 1992, pp. 517–520.

[8] R. Kneser, H. Ney, Improved backing-off for m-gram language modeling, in: International Conference on Acoustics, Speech, and Signal Processing, Vol. 1, IEEE, 1995, pp. 181–184.

[9] Z. E. Imel, M. Steyvers, D. C. Atkins, Computational psychotherapy research: Scaling up the evaluation of patient–provider interactions, Psychotherapy 52 (1) (2015) 19–30.

[10] A. Stolcke, Srilm — an extensible language modeling toolkit, in: Proc. Interspeech, 2002.

[11] A. J. Smola, B. Schölkopf, A tutorial on support vector regression, Statistics and computing 14 (3) (2004) 199–222.

[12] C.-C. Chang, C.-J. Lin, Libsvm: a library for support vector machines, ACM Transactions on Intelligent Systems and Technology (TIST) 2 (3) (2011) 27.
